# Reconstruction of the respiratory signal through ECG and wrist accelerometer data

**DOI:** 10.1038/s41598-020-71539-0

**Published:** 2020-09-03

**Authors:** Julian Leube, Johannes Zschocke, Maria Kluge, Luise Pelikan, Antonia Graf, Martin Glos, Alexander Müller, Ronny P. Bartsch, Thomas Penzel, Jan W. Kantelhardt

**Affiliations:** 1 Institute of Physics, Martin-Luther-University Halle-Wittenberg, 06099 Halle, Germany; 2 Institute of Medical Epidemiology, Biostatistics and Informatics, Faculty of Medicine, Martin-Luther-University Halle-Wittenberg, 06099 Halle, Germany; 3 Interdisziplinäres Schlafmedizinisches Zentrum, Charité - Universitätsmedizin Berlin, 10117 Berlin, Germany; 4 Klinik und Poliklinik für Innere Medizin I, Technische Universität München, 81675 Munich, Germany; 5 Department of Physics, Bar-Ilan University, Ramat Gan, 5290002 Israel; 6 Saratov State University, Saratov, Russia

**Keywords:** Respiratory signs and symptoms, Signal processing, Time series

## Abstract

Respiratory rate and changes in respiratory activity provide important markers of health and fitness. Assessing the breathing signal without direct respiratory sensors can be very helpful in large cohort studies and for screening purposes. In this paper, we demonstrate that long-term nocturnal acceleration measurements from the wrist yield significantly better respiration proxies than four standard approaches of ECG (electrocardiogram) derived respiration. We validate our approach by comparison with flow-derived respiration as standard reference signal, studying the full-night data of 223 subjects in a clinical sleep laboratory. Specifically, we find that phase synchronization indices between respiration proxies and the flow signal are large for five suggested acceleration-derived proxies with $\gamma = 0.55 \pm 0.13$ for males and $0.58 \pm 0.14$ for females (means ± standard deviations), while ECG-derived proxies yield only $\gamma = 0.36 \pm 0.16$ for males and $0.39 \pm 0.14$ for females. Similarly, respiratory rates can be determined more precisely by wrist-worn acceleration devices compared with a derivation from the ECG. As limitation we must mention that acceleration-derived respiration proxies are only available during episodes of non-physical activity (especially during sleep).

## Introduction

There is substantial evidence that deviations of the respiratory rate from its normal behavior can be used as a predictor of clinically relevant and potentially fatal events and conditions (see, e.g., the very recent review paper by Liu et al.^1^ and references therein), although the relevance of respiratory rate has long been overlooked in clinical setting^2^ and other fields^3^. For example, spontaneous breathing rates below six breath per minute (bpm) was prospectively shown to be a stronger predictor of subsequent in-hospital mortality than abnormal heart rate, hypertension or the decrease (or loss) of consciousness^4^. In a very recent study of non-invasive risk assessment for cardiac patients, abnormally high respiratory rate ($> 18.6 \, \hbox {bpm}$) and low expiration-triggered sinus arrhythmia turned out to be among the three most sensitive early risk indicators as components of the Polyscore index^5^; previous work also demonstrated the importance of respiratory rate for cardiac patients^6,7^. Therefore, it is appropriate to include measurements of respiratory rate and the influence of respiration activity on the heart in large cohort studies that aim at identifying early indicators for health risks and to study effects of healthy aging^8,9^.

Although many methods and technologies for the measurement of respiratory rate and the identification of breathing intervals have been suggested over the past decades^1,10,11^, there is still a need for inexpensive, reliable, and non-obtrusive sensors. In order to assess respiratory behavior in large epidemiological cohort studies with many thousands of participants from the general population, the handling of the measurement technology should be as easy as possible with a minimum of additional costs. The derivation of respiration proxies from the recordings of devices already used in such studies are thus particularly valuable. An important approach in this regard is exploiting the respiratory modulation of other physiological signals, such as the electrocardiogram (ECG) often registered in long-term (Holter) recordings for 24 h^1,12–14^ or during sleep studies^15^. ECG amplitude and baseline as well as frequency are modulated by respiration via motions of the heart axis and respiratory sinus arrhythmia (RSA), respectively, leading to more than a dozen of respiration proxies^12^.

In addition, similar proxies can be derived from the photoplethysmogram (PPG)^16,17^, with best signals recorded at the forehead and the finger for normal and deep breathing pattern, respectively^18^. In a systematic comparison study, feature-based techniques in the time domain turned out to be generally superior to filter-based techniques and techniques in the frequency domain^1,12^. In addition, feature-based time-domain techniques facilitate the determination of the individual breathing intervals (instead of a mere breathing rate), and they are more useful for studying the data of patients with possibly irregular breathing (e.g., due to apneas) or extremely low or high breathing rates.

A few approaches tried a fusion of ECG or PPG derived respiration with respiration proxies from accelerometer and gyroscope data^19–21^. Accelerometer-based methods for measuring breathing-related movements have been roughly validated^22–24^. However, mainly accelerometers and gyroscopes, appropriately positioned over the diaphragm^1^, the dome-shaped skeletal muscle of the thoracic cavity^25^ (or the chest wall^11^), as well as the forehead^26^ have been considered, so that an additional sensor is needed in these efforts.

In this paper we propose and validate an approach for extracting proxy signals for respiratory events from wrist accelerometer data. Wrist accelerometers are often employed in large cohort studies for the purpose of activity/inactivity tracking as well as sleep/wake identification of the subjects. No cables nor obtrusive sensors are needed, since a wrist accelerometer is worn like a common wrist watch. There are a few previous studies on wrist accelerometer data^10,26,27^ that focus on estimating mean respiratory rate using spectral techniques.

## Results

Here, we present our results for respiratory proxies derived from wrist accelerometer data. Specifically, we consider the instantaneous respiratory phases and respiratory rates derived from acceleration recorded for all three perpendicular axes (*x*, *y*, and *z*) on the non-dominant arm of 223 subjects. For details, we refer to the “Methods” section and Tables 1 and 2, in particular. In addition to the proxies ${\hbox {Acc}}_x$, ${\hbox {Acc}}_y$, and ${\hbox {Acc}}_z$ for the normal axes, we have studied data for the corresponding rotational angles $\vartheta$ and $\varphi$ of the wrists.

**Table 1:** List of respiration proxies considered in this work; see “Methods” section below for the description of the signals and particularly Fig. 6 for wrist acceleration measurements.

Measure	Description
${\hbox {Acc}}_x$	Wrist acceleration in longitudinal direction (in mg)
${\hbox {Acc}}_y$	Wrist acceleration in lateral direction (in mg)
${\hbox {Acc}}_z$	Wrist acceleration in lateral direction (in mg)
$\vartheta$	rotational angle of the wrist (in rad)
$\varphi$	rotational angle of the wrist (in rad)
B1	Average of maximum and minimum of QRS complex (in $\mu \hbox {V}$)
B2 (EDR)	Difference of maximum and minimum of QRS complex (in $\mu \hbox {V}$)
B3	Duration of RR interval (in ms)
B5	Maximum of QRS complex (in $\mu \hbox {V}$)

In order to relate with previous literature, we compare our results with respiratory proxies derived from ECG recordings. Specifically, we have considered the following four previously established ECG-derived proxies of respiration: averages of maximum and minimum of QRS complex (B1), differences of maximum and minimum of QRS complex [B2, also commonly referred to as ECG-derived respiration (EDR)], duration of RR intervals (B3), and maxima of QRS complex (B5), see also Table 1 and “Methods” section. We have selected these four proxies based on their superior performance in a previous study^13^.

**Table 2:** Overview of all subjects included in the analysis.

Diagnosis	Females	Males
No diagnosed sleep disorder	5	6
Sleep-related breathing disorders (SRBD)	42	69
Insomnia	29	15
Central disorders of hypersomnolence	25	16
Sleep-related movement disorder	20	11
Parasomnias	6	5
Circadian rhythm sleep-wake disorders	0	6
Other sleep disorders	5	7
All subjects	110	113

Subjects with multiple diagnoses are counted in each appropriate diagnosis line, i.e., multiple times. The last line reports data for all subjects irrespective of diagnosis.

### Phase synchronization

**Figure 1:**
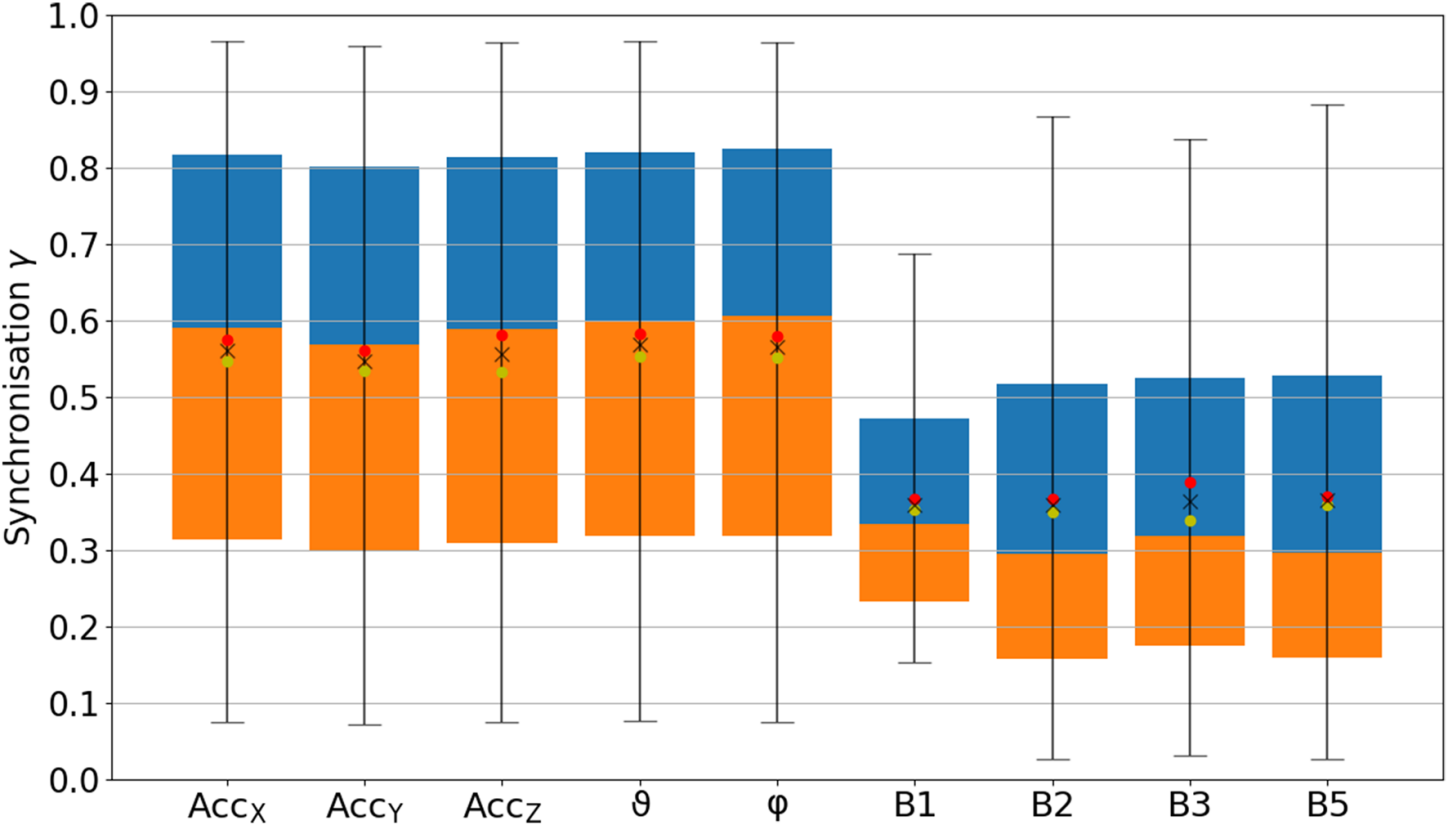
Boxplot of the average synchronization between proxies and measured respiration. Phase synchronization indices $\gamma$ according to Eq. (5) between all respiration proxies (see Table 1) and the recorded flow signal have been averaged over the complete sleeping period and all 223 subjects (see Table 2). The orange part of each box represents the values between the lower quartile and the median, and the blue part represents the values between the median and the upper quartile. The ends of the whiskers mark the 2.5% quantile and the 97.5% quantile, respectively. The total average values appear as black crosses in the boxplot along with the averages for male (yellow dots) and female (red dots) subjects. According to t-tests, the results for ${\hbox {Acc}}_x$, ${\hbox {Acc}}_y$, ${\hbox {Acc}}_z$, $\vartheta$, and $\varphi$ were significantly different from all other results ($p < 0.001$), but not significantly different from each other. The same holds for the results regarding the ECG-derived proxies B1, B2, B3 and B5. Differences between males and females were marginally significant ($0.05 > p \ge 0.01$) for ${\hbox {Acc}}_x$, ${\hbox {Acc}}_y$, $\varphi$, $\vartheta$, and B2, and significant ($p < 0.01$) for ${\hbox {Acc}}_z$ and B3.

Figure 1 shows a boxplot of the phase synchronization indices $\gamma$ [Eq. (5)] for all considered respiration proxies (see Table 1) compared to flow as the respiration standard signal, including the results for all 223 subjects (see Table 2). Although many previous works focused on EDR proxies (see “Methods”), we found that all wrist acceleration proxies perform significantly better (t-tests: $p < 0.001$). The rotational angles $\varphi$ and $\vartheta$ performed best with averages $\gamma = 0.55 \pm 0.13$ ($0.58 \pm 0.14$) and $0.55 \pm 0.13$ ($0.58 \pm 0.15$) for males (females), respectively (mean ± standard deviation). ${\hbox {Acc}}_x$ ($\gamma = 0.55 \pm 0.13$ and $0.58 \pm 0.15$ for males and females, respectively), ${\hbox {Acc}}_y$ ($\gamma = 0.53 \pm 0.13$ and $0.56 \pm 0.13$), and ${\hbox {Acc}}_z$ ($\gamma = 0.53 \pm 0.13$ and $0.58 \pm 0.15$) also achieved significantly larger average synchronization indices than each of the ECG-derived proxies. The best ECG proxies were B3 (based on RSA) with $\gamma = 0.34 \pm 0.12$ ($0.39 \pm 0.14$) and B5 with $\gamma = 0.36 \pm 0.12$ ($0.37 \pm 0.18$) for males (females). Differences between males and females were significant for ${\hbox {Acc}}_z$ ($p<0.05$) and B3 ($p<0.01$).

**Figure 2:**
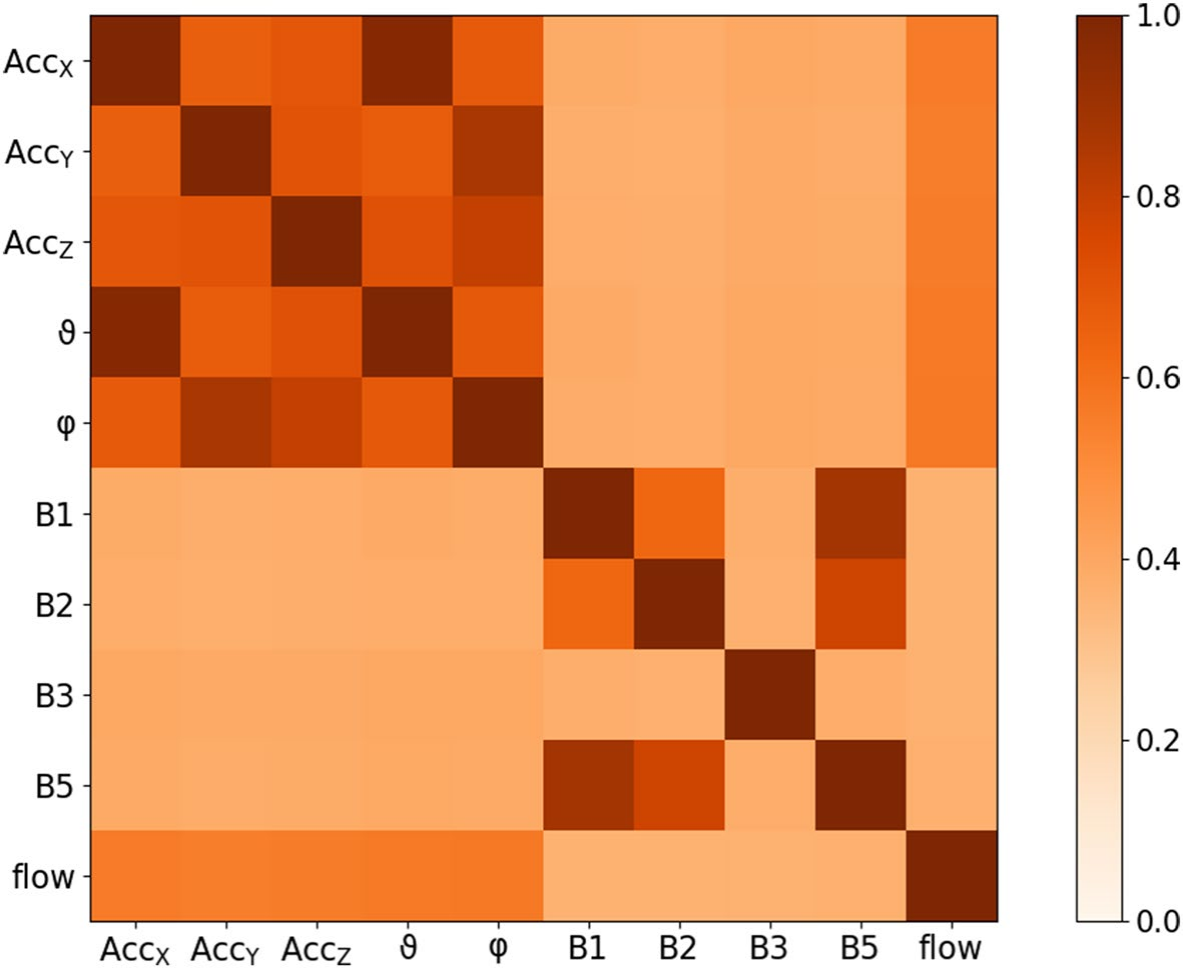
Synchronization matrix. Phase synchronization indices $\gamma$ according to Eq. (5) between all pairs of respiration proxies (see Table 1) and the flow have been averaged over the complete sleeping period and all 223 subjects. They are presented in a symmetrical color-coded matrix with brown indicating full synchronization and white indicating no synchronization—see color bar on the right.

A direct comparison of all subjects with sleep-related breathing disorders (SRBD) and all other subjects yielded similar results for both subgroups, although, in the SRBD subgroup, the $\gamma$ values were significantly ($p<0.05$) smaller for all acceleration-derived proxies and even reached $p<0.01$ for $\vartheta$ and ${\hbox {Acc}}_z$. ECG-derived proxies B1 and B5 yielded slightly larger $\gamma$ values in the SRBD subgroup ($p<0.05$), while differences were not significant for B2 and B3.

**Figure 3:**
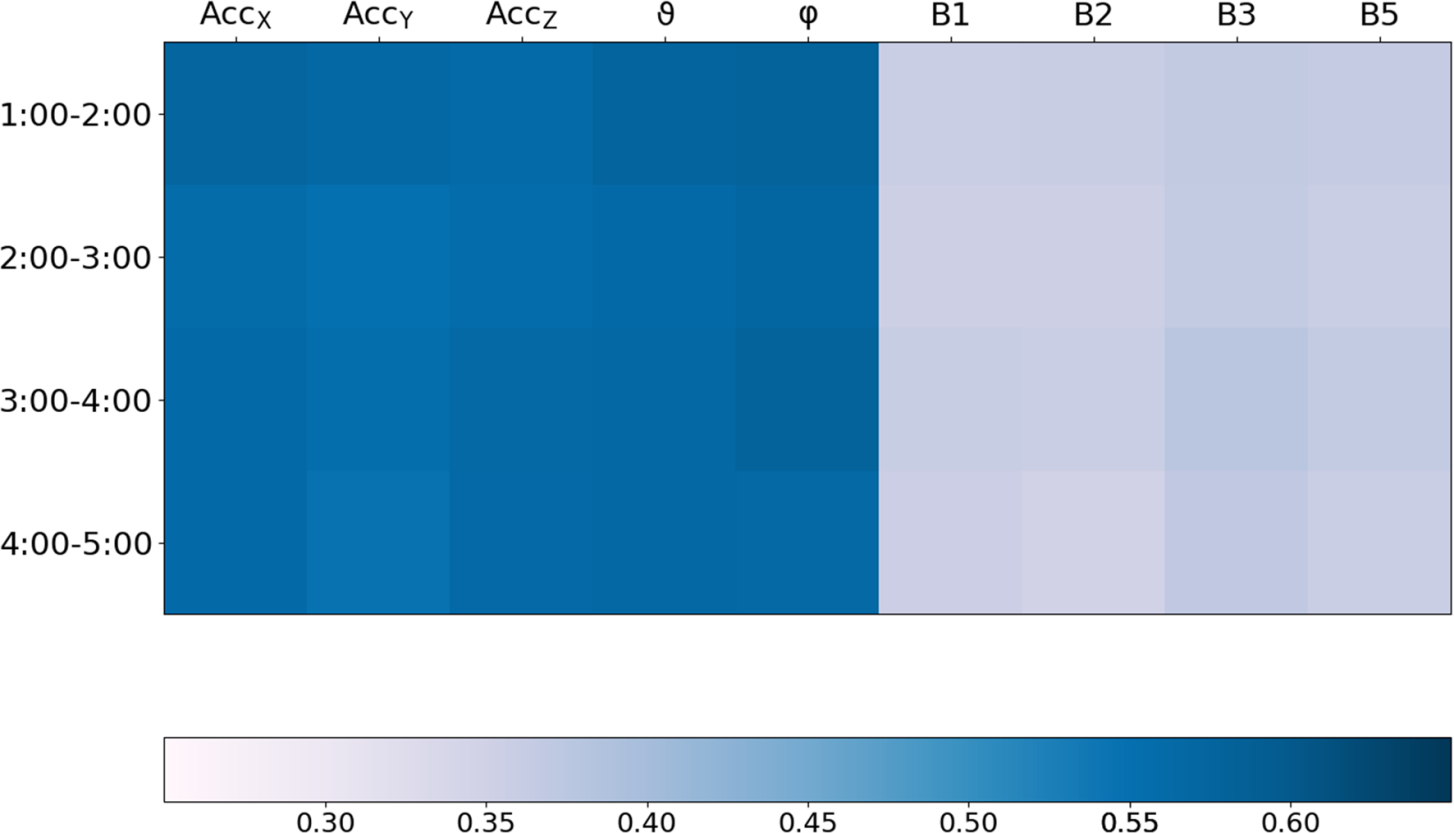
Best synchronization with flow during nocturnal hours. For each respiration proxy (see Table 1), this matrix shows the average phase synchronization index $\gamma$ with respect to the flow signal (see color bar on the bottom) during the considered nocturnal hour.

Figure 2 shows the mean values of all pairwise synchronization indices $\gamma$ between the proxies and the flow. Clearly, all acceleration-derived proxies are quite well synchronized to each other. $\vartheta$ is very similar to ${\hbox {Acc}}_x$, while $\varphi$ is similar to ${\hbox {Acc}}_y$ and ${\hbox {Acc}}_z$, for example [cp. Eq. (2)]. Many ECG-derived proxies (particularly B1, B2 and B5) are well synchronized with each other, but not so well synchronized with the recorded respiratory flow.

**Figure 4:**
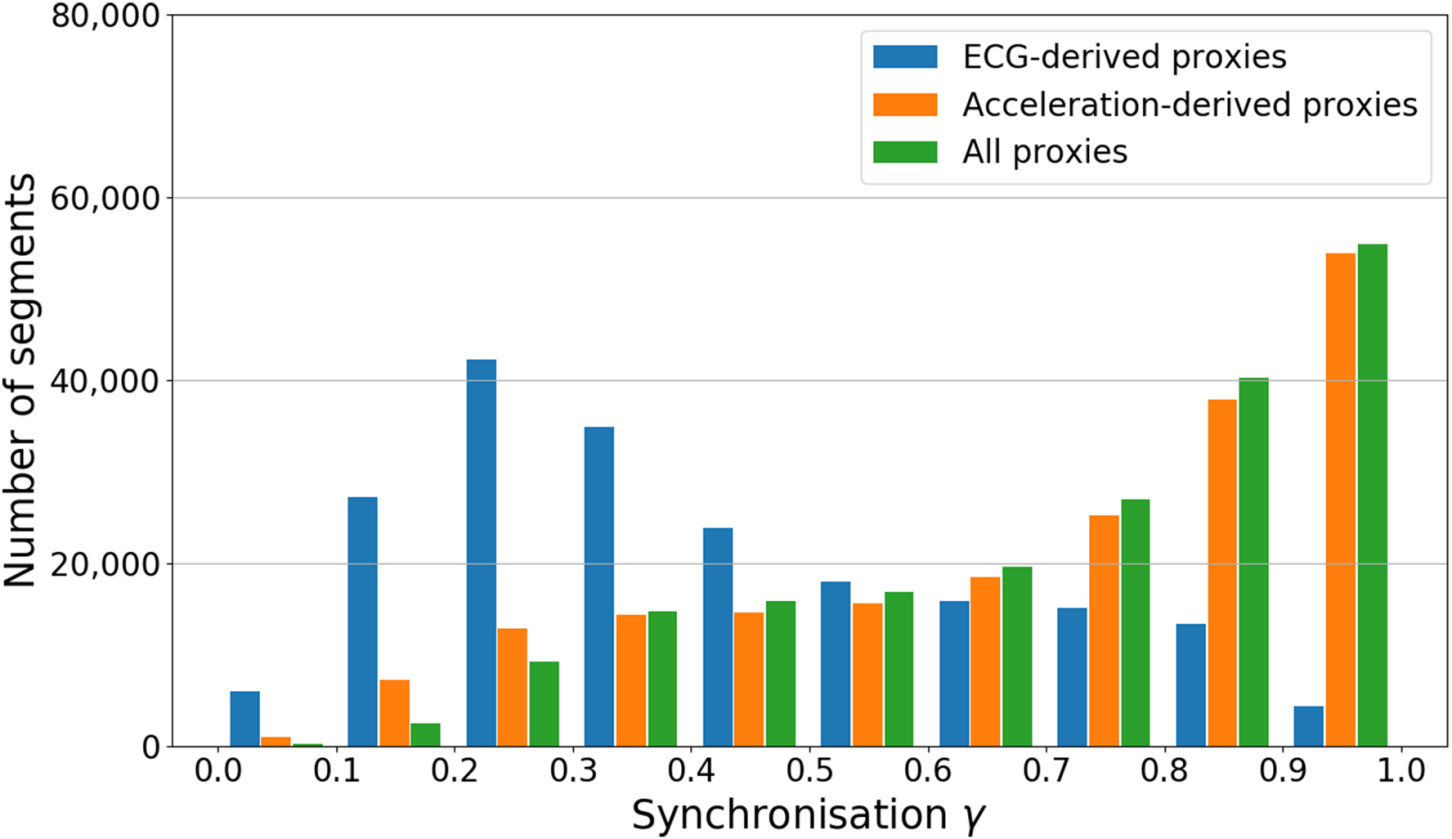
Histogram of best achievable proxy synchronization. The histograms show the numbers of 30 s segments of the total data (all 223 subjects) for which a $\gamma$ value in the particular interval (0.0 to 0.1, 0.1 to 0.2, etc.) could be achieved taking into account all proxies (green), only acceleration-derived proxies (orange), and only ECG-derived proxies (blue).

**Figure 5:**
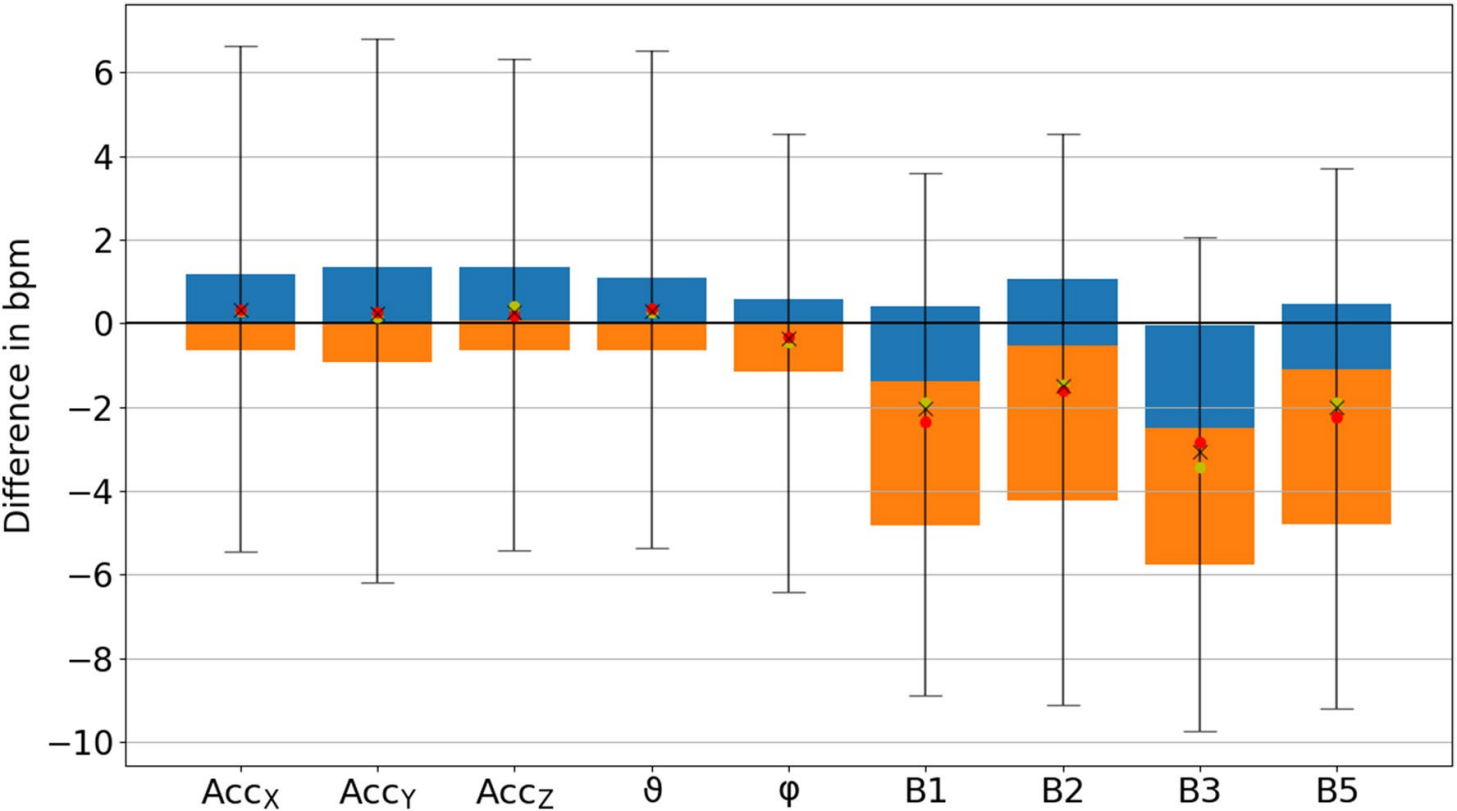
Boxplot of average difference of respiratory rate. The differences of respiratory rates calculated from each respiration proxy and the measured flow signal has been averaged over all 30 s segments and all 223 subjects. The boxes and markers correspond to those in Fig. 1. Note that $\varphi$ yielded a slightly lower respiratory rate (negative difference) than the other acceleration-derived proxies. The results regarding the ECG-derived proxies B1, B2, and B5 were also lower. Differences between males and females were not significant.

In Fig. 3 the phase synchronization of all proxies to the recorded flow signal is traced for several nocturnal hours with least wakefulness (1:00 am to 5:00 am). Overall, $\varphi$ and ${\hbox {Acc}}_x$ yielded the best synchronization, but the differences compared with $\vartheta$, ${\hbox {Acc}}_y$, and ${\hbox {Acc}}_z$ are tiny. It seems that the synchronization of B3 slightly increases with time.

**Figure 6:**
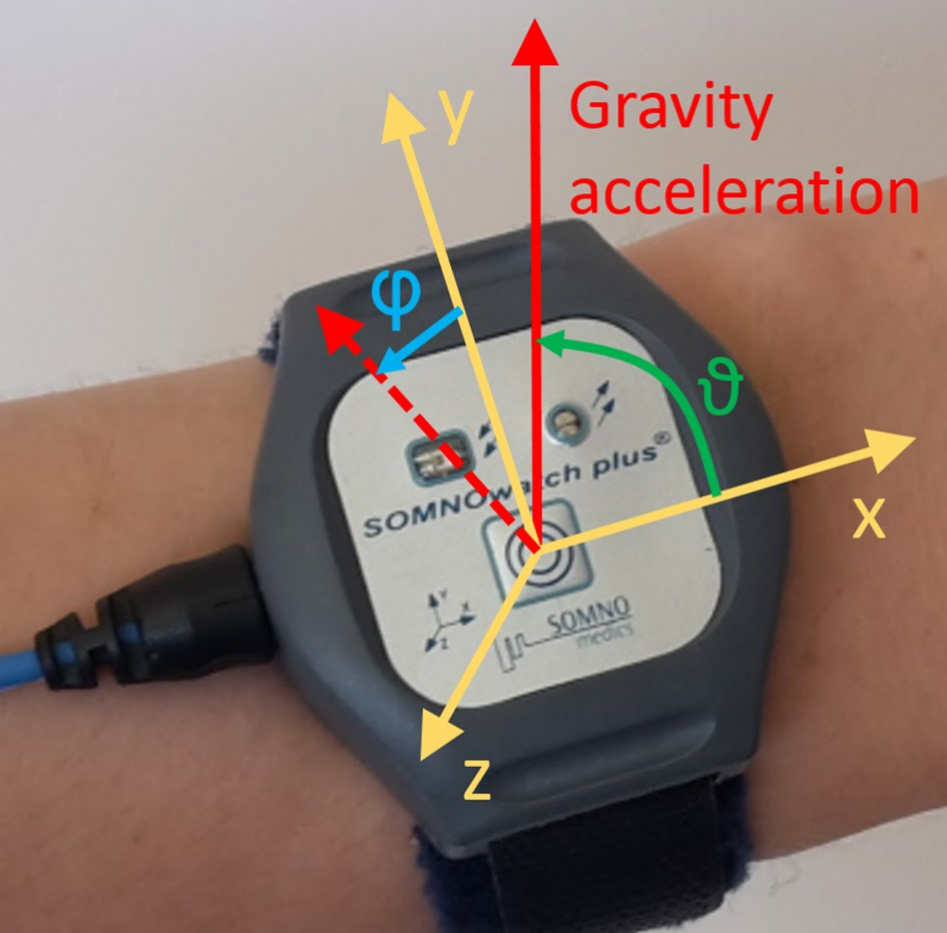
Acceleration recording at the wrist. The photo shows the placement of the SOMNOwatch plus device (Somnomedics GmbH, Randersacker, Germany) at the wrist with the coordinate axes (*x*, *y*, and *z*; yellow) according to the device’s orientation as well as the gravity acceleration vector (red) pointing vertically upwards from the center of the earth. The device measures the three components of the gravity acceleration with respect to its coordinate axes. From this data the two orientational angles, $\vartheta$ = angle between *x* axis and gravity acceleration and $\varphi$ = angle between *y* axis and projection of the gravity acceleration into the $y-z$ plane (dashed red vector), can be calculated according to Eq. (3).

Figure 4 shows the distributions of synchronization indices for all 30 s segments of all recordings. If only ECG-derived proxies were available for the selection, synchronization indices between 0.2 and 0.3 would be most common, and values above 0.9 could rarely be achieved. However, for accelerometer data derived proxies, synchronization indices above 0.9 turned out to be the most frequent. In fact, the distributions of $\gamma$ values reached if all proxies are considered is not much different from the distribution achieved for acceleration-derived proxies only, except in the regime of $\gamma < 0.2$.

**Figure 7:**
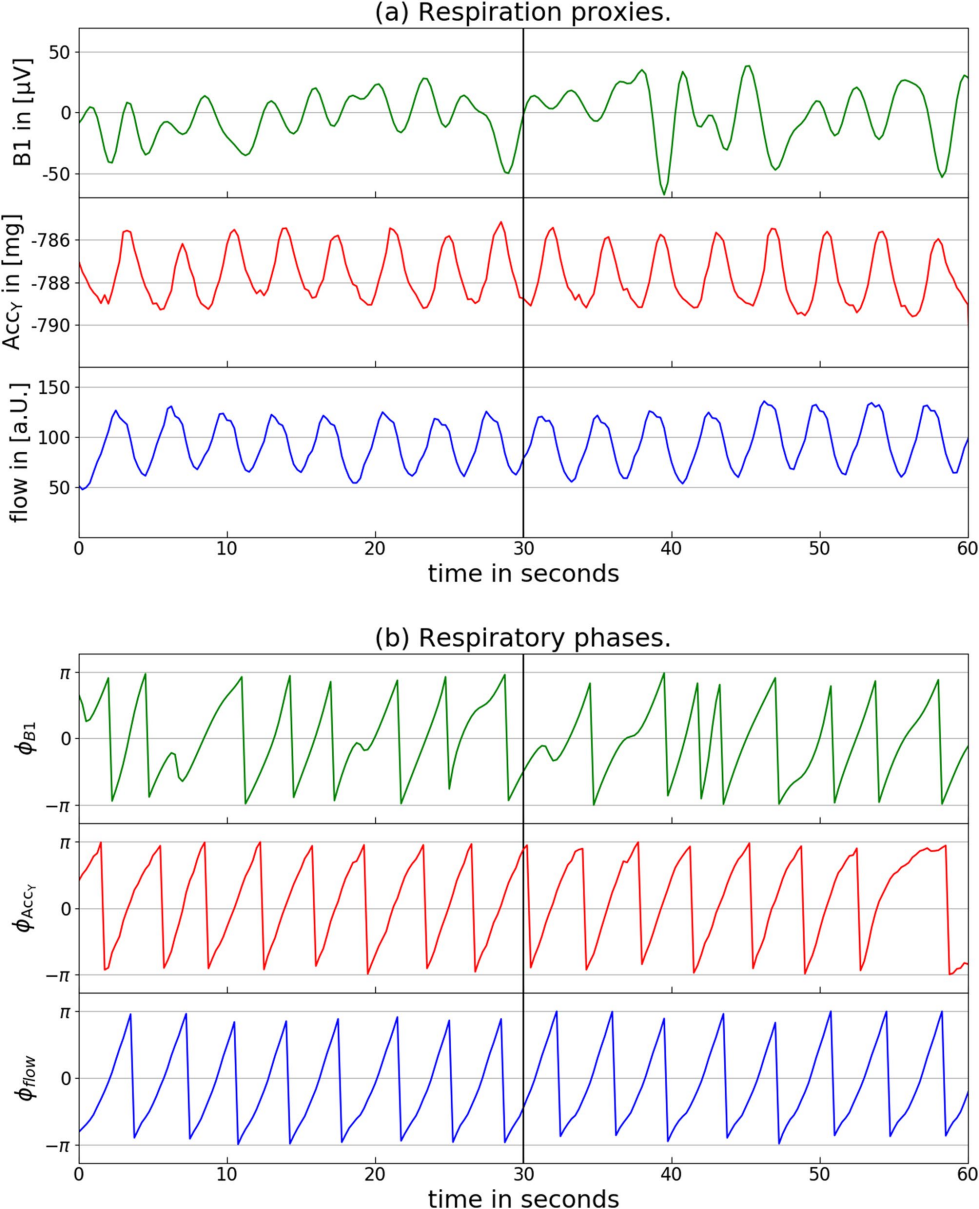
Proxy data and reconstructed respiratory phases. (**a**) Respiration proxies determined from the averages of maximum and minimum of each QRS complex (proxy B1, green) and smoothened lateral *y* axis acceleration ${\hbox {Acc}}_y$ (red) recorded at the subjects wrist during sleep. For comparison, the respiratory flow recorded by a separate sensor placed at the subject’s nose is also shown (blue). The vertical line at $t=30 \hbox {s}$ marks the window size we have used for our comparisons. (**b**) Corresponding respiratory phases $\phi (t)$ derived from each of the signals shown in part (**a**) via Hilbert transform and Eq. (4).

### Respiratory rates

Figure 5 shows the differences of average respiratory rates derived from all proxies and the recorded flow signal. The acceleration-derived respiratory rates closely agreed with the real respiratory rate with average deviations between $-0.38$ breaths per minute (bpm, $-2.6$%) for $\varphi$ and up to + 0.32 bpm (+ 2.1%) for the other acceleration-derived proxies. The ECG-derived proxies generally underestimated respiratory rates with average deviations between $-3.1 \, \hbox {bpm}$ and $-1.5 \, \hbox {bpm}$ ($-21.2$ to $-10.4$%). However, we would like to stress that the estimation of mean respiratory rates is not the main goal of our medically oriented approach, which shall also capture interruptions of respiration (apneas) as well as times with very low and high respiratory rates, i.e., extreme events and respiratory variability. In this respect, we would like to note that significant differences between subjects with and without SRBD (present in the flow signal) could be identified in most acceleration-based proxies (${\hbox {Acc}}_x$, ${\hbox {Acc}}_y$, $\vartheta$, and $\varphi$) and in B1, but not in B2, B3, or B5.

## Discussion

In this paper, we have introduced and validated an approach for obtaining respiration proxies from nocturnal long-term wrist acceleration measurements from 223 clinical subjects including (but not restricted to) patients with various sleep-related disorders. We have shown that each of the the suggested five acceleration-derived proxies is significantly ($p<0.001$) more reliable than each of the four known standard ECG-derived respiration proxies, exploiting ECG baseline, amplitude, and frequency changes.

For comparison, we have considered four established ECG-derived proxies of respiration, B1, B2, B3, and B5, selected because of their superior performance in the study by Charlton et al.^13^, where 15 proxies for a reconstruction of respiratory activity from one-channel ECGs were compared in healthy subjects. Measures B1, B2, and B5 are based on the varying direction of the heart axis during the respiratory cycle, leading to ECG baseline wander (exploited in B1 and B5) and ECG amplitude modulation (exploited in B2 and B5). B2 is also known as ECG-derived respiration (EDR) method and considered in many other studies, see e.g.^28^ B3 is based on the effects of RSA^29^ leading to ECG frequency modulation and thus also known as RSA method^30^. Specifically, in the study of Charlton et al., B2 yielded the highest median subject-specific correlation coefficients (CC) with respiratory activity in both, young and elderly subjects (CC = 0.76 and 0.77, respectively), B5 performed similarly well (CC = 0.74 and 0.76, respectively), and B1 was at the third rank for elderly subjects (CC = 0.72, versus 0.66 in the young). We also included the best frequency modulation based proxy B3 for comparison, although it performed well in young subjects only (CC = 0.66, versus 0.44 in the elderly)^13^.

The variation of phase synchronization values among the subjects was large for the acceleration-derived $\gamma$ values as indicated by the widths of the box plots and their whiskers in Fig. 1. This is most probably due to multiple possibilities of arm placement of the subjects during sleep. While an arm lying on the chest will lead to an improved respiration proxy, a widely extended arm leads to weaker respiratory movements at the wrist. As expected, the length of the acceleration vector, *r*, reached a drastically lower phase synchronization index (not shown in Fig. 1), since the magnitude of the gravitational force does not change with respiration. Nevertheless, the directions of this vector in the reference frame of the wrist-fixed recording device change as respiration causes slight repetitive turns of the wrist^1^, as exploited in our other accelerometer-derived proxies. While B2 was the best proxy in earlier studies^13^, in our study all ECG-derived respiration proxies yielded similar (not significantly different) results.

The respiratory rate can be determined more exactly by wrist worn acceleration devices. Respiration proxies obtained from the data of all three accelerometer axes using a simple moving average smoothening (${\hbox {Acc}}_x$, ${\hbox {Acc}}_y$, ${\hbox {Acc}}_z$) as well as the derived rotation angle $\vartheta$ turned out to be similarly reliable. Since the derived rotation angle $\varphi$ yielded a significant deviation in the respiratory rate, we suggest not using it although the approach seemed very promising initially. Based on our data, our recommendation goes to ${\hbox {Acc}}_x$ and $\vartheta$.

We note that recent work on a BioWatch^26^ used single-axis wrist accelerometer data in the frequency domain from 0.13 to 0.66 Hz (corresponding to 8 and 40 breaths per minute) to estimate respiratory rates. The technique focused on average breathing rate in intervals of 20 s as determined via spectral analysis, not trying to identify individual breaths, breathing interruptions, or breath-to-breath intervals. Besides that, it was limited to 32 h of sleep data from three subjects and 72 mins of daytime data (sitting, standing and lying without motion) from twelve subjects. Another recent study determined the average respiratory rates in 15 subjects using wrist accelerometer data^27^, reporting an average deviation of 16.6% with respect to respiratory rate from a chest band. Another paper from the same group reported that respiratory rate can be most reliably estimated via accelerometry, if the sensor is attached to the subjects’ torso or shoulders^31^.

As an improvement compared to BioWatch, the recently introduced SleepMonitor^10^ exploited wrist accelerometer data recorded at 16 Hz in 30-s windows, fusing the spectrally determined respiratory rates from all three axes and this way obtaining results with errors about half as large as those of BioWatch. Mean absolute errors for the wrist-motion determined respiratory rate as compared with the chest-motion determined rate were 0.72 and 1.08 breaths per minute for normally and disorderedly (either sleep apnea or intentional strong breathing variations) breathing subjects, respectively. The technique, validated with data from 70 nights of 16 subjects (including two subjects with sleep-disordered breathing), included a Kalman filter working with predictions of respiratory rate in addition to FFT filtering and did not try to capture interruptions of respiration (apneas) nor extreme variations of respiratory rates.

Since our approach fully works in the time domain, not involving spectral analysis, it is rather insensitive to non-stationarities of the recorded data and not limited to certain ranges of respiratory rate or requiring smooth changes of respiratory activity. Therefore, not only respiratory rate but also possibly clinically relevant extreme events and interruptions of respiratory activity can be assessed. Although further development, optimization, and validation is necessary before our approach could be clinically applied, we think our method can be used in its current form to derive the respiratory signal from nocturnal accelerometer recordings obtained in large cohort studies. Such cohorts are currently recorded in the framework of, e.g., the UK Biobank study and the German National Cohort (GNC) study.

### Limitations

We must certainly mention that acceleration-derived respiration proxies are available during episodes of non-physical activity (especially during sleep) only, while ECG-derived respiration is not limited in this way. We also remark that our approach for using accelerometer data as a respiratory proxy will fail in a zero-gravity environment, e.g. in a space station, since it requires the vertical gravitational direction as reference.

## Methods

### Measurements

All measurements took place in the sleep laboratory of the Charité-Universitätsmedizin Berlin, Germany, between April 2017 and December 2018. The study was approved by the ethics committee of the Charité-Universitätsmedizin Berlin and registered at the German Clinical Trial Register (DRKS) with ID DRKS00016908. All methods were performed in accordance with the relevant guidelines and regulations. In total, 392 subjects were included in the study after signing informed consent. During their first diagnostic night at the sleep laboratory, all subjects wore a SOMNOwatch plus device (Somnomedics GmbH, Randersacker, Germany), recording simultaneously 3d wrist acceleration of the non-dominant arm at 128 Hz sampling rate (see Fig. 6) and a one channel ECG at 256 Hz. Furthermore, full polysomnography (PSG, including recordings of electroencephalography (EEG), electrooculography (EOG), electromyography (EMG), ECG, respiratory flow, etc.) was recorded using either the system Alice (Löwenstein Medical, Bad Ems, Germany), Embla (Embla systems, Broomfield, CO, United States), or SOMNOscreen (Somnomedics GmbH, Randersacker, Germany). For our analysis we used the acceleration and ECG data recorded by SOMNOwatch and the respiratory flow signal recorded by the PSG system.

The measurements of the SOMNOwatch device and the PSG system were synchronized after recording by matching the R peaks of the ECGs recorded by both of them. 145 subjects had to be excluded from further analysis, since reliable synchronization could not be established this way because of poor ECG quality in either of the two recordings. We note that this synchronization method required matching *R* peaks from the simultaneously recorded ECGs during each 20 minutes of the recordings, since we identified jumps (i.e. unmarked time gaps) in more than 15% of all PSG recordings across all three recording systems; no such gaps occurred in the Somnowatch recordings. In addition, we had to determine and take into account drifts of the recorded time cumulating to typically 1–2 s per night. Another 24 subjects had to be excluded because their respiratory recordings from the SOMNOscreen system could not be successfully exported into the European data format (EDF+) leading to completely or substantially (for more than half of the recording time) missing flow signals. The final 223 subjects, aged between 18 and 78 years (mean $47.9~ \pm ~13.7 \, \hbox {years}$) with average body mass index $27.7~ \pm ~5.7~ {\hbox {kg/m}}^2$, had an average time in bed (TiB) of $7.6~ \pm ~0.8~ \hbox {h}$. Only data recorded during the lights-off period were considered. All subjects were regular patients of the sleep laboratory with confirmed sleep disorders. In Table 2 we list the frequency of sleep disorders classified by ICSD-3 (International Classification of Sleep Disorders).

### Reconstruction of respiratory signals from accelerometry

Our initial observation of a peak in the 0.3 Hz range (corresponding to $\approx 18$ breaths per minute) in nocturnal three-axis accelerometry data recorded at the wrist^32^ was the starting point for our approach towards respiration proxies. After we had found that the high amplitude resolution of modern accelerometers (down to $3 \, \hbox {mg} \, \approx 0.03 \, {\hbox {m/s}}^2$) can resolve tiny motions caused by respiratory activity^10,32,33^, we have systematically studied if this effect can be used for a practical derivation of respiration proxies.

The data processing consists of several steps and starts by smoothing the recorded 128 Hz raw acceleration data $\ddot{x}(t)$ by calculating a moving average. Specifically, we calculated a moving average with a window width of $\pm 0.5$ s (i.e., $\pm 64$ data points),

1 $$\begin{aligned} {\text{Acc}}_x(t) = {1 \over 129} \sum _{j=-64}^{+64} \ddot{x}(t+(j/128 {\text{Hz}})) \end{aligned}$$

to obtain smoothened longitudinal acceleration (with the *x* axis in the direction towards the elbow and the hand, see Fig. 6) as first respiration proxy. Similarly, we obtained smoothened lateral accelerations for the the *y* and *z* axes oriented perpendicular to *x*, ${\hbox {Acc}}_y$ and ${\hbox {Acc}}_z$, see Table 1. The window size of 1 s in Eq. (1) was chosen such that there is typically one heartbeat in each window so that effects of heartbeats and pulse wave propagation through the wrist (see also^34^) are systematically dampened in this moving average procedure. Finally, a resampling to a rate of 4 Hz is applied, since respiration proxies do not need temporal resolutions beyond that.

The red curve in Fig. 7a shows an example for the final respiration proxy determined from *y* axis accelerometry during sleep. The respiratory cycles can clearly be identified.

We note however, that wrist accelerations caused by respiratory activity are relatively small. In fact, what the acceleration measurement device registers is not an respiration-caused acceleration per se, but instead a slight turning of the wrist in synchrony with the respiratory activity (see also^1^). This turning leads to slightly modified projections of the (vertical) gravitational force onto the axes of the coordinate system of the sensor device and thus to slight variations of the *x*, *y* and *z* components of the registered gravitational vector, see Fig. 6. Therefore, one can expect that one or both of the two angles representing the direction of the gravitational vector are even better proxies for respiration than the components in particular directions. Hence, in addition to ${\hbox {Acc}}_x$, ${\hbox {Acc}}_y$ and ${\hbox {Acc}}_z$, we consider their angles in spherical coordinates, $\vartheta$ (angle between gravity vector and *x* axis), and $\varphi$ (angle between projection of gravity vector into the $y-z$ plane and *y* axis) as shown in gravity vector Fig. 6:

2 $$\begin{aligned} {\mathrm{Acc}}_x = r \cos \vartheta , {\mathrm{Acc}}_y = r \cos \varphi \sin \vartheta , {\mathrm{Acc}}_z = r \sin \varphi \sin \vartheta , \end{aligned}$$

corresponding to

3 $$\begin{aligned} r = \sqrt{{\mathrm{Acc}}_x^2+{\mathrm{Acc}}_y^2+{\mathrm{Acc}}_z^2}, \varphi = \arctan _2({\mathrm{Acc}}_z,{\mathrm{Acc}}_y), \vartheta = \arccos ({\mathrm{Acc}}_x/r), \end{aligned}$$

see also Table 1. We have applied the same smoothening [Eq. (1)] and resampling procedure to *r*, $\varphi$, and $\vartheta$ as to the acceleration components above. We expect that $\varphi$ and/or $\vartheta$ are much better respiration proxies than *r* if our assumption regarding changing directional projections of the (constant) gravitational vector holds. Hence, the suggested transformation can facilitate the selection of an optimal proxy.

Finally, instantaneous respiratory phases have been calculated from all respiration proxies as well as the directly registered respiratory signal flow(*t*) (blue curve in Fig. 7a). The first step in this procedure was the normalization of the time series via (i) subtraction of a moving average similar as in Eq. (1) and (ii) division by a moving standard deviation. Both of these moving quantities have been calculated for windows of $\pm 5$ s duration around the center point, so that effectively frequencies between 0.1 Hz (cutoff by moving average with 10 s window size) and 1 Hz (cutoff by moving averag with 1 s window size) remain in the respiration proxies. The resulting narrow-banded signals oscillating around zero can easily be transformed into instantaneous respiratory phases $\phi (t)$ via a Hilbert transform^35^,

4 $$\begin{aligned} x(t) + i \mathrm{HT}[x(t)] = A(t) \exp [i \phi _x(t)], \end{aligned}$$

using $\phi _x(t) = \arctan _2(\mathrm{HT}[x(t)], x(t))$ for $x={\mathrm{Acc}}_x$, ${\hbox {Acc}}_y$, …, flow(*t*). Fig. 7b shows these reconstructed respiratory phases for all signals of Fig. 7a.

In this study we focus on analyzing and comparing instantaneous respiratory phases (instead of respiratory rates or breathing cycles), because the phases comprise all information without the need to define certain points in the breathing cycle, e.g., beginning or ending, transition form inspiration to expiration, etc. Respiratory phases increase continuously from $-\pi \approx -3.14$ to $+\pi$ and then jump back to $-\pi$ in a sawtooth-like pattern, see Fig. 7b. However, since phases are actually defined on a circle, the values of $-\pi$ and $+\pi$ refer to identical phase angles, the selection of the jump point is arbitrary, and constant phase shifts (possibly including multiples of $2 \pi$) have no relevance. Therefore, when comparing instantaneous phase signals, their differences are always taken modulo $2 \pi$, and constant differences are disregarded. This is advantageous, since proxies derived, e.g., from inverted flow or acceleration (or ECG) signals, leading to phases differing by $+\pi$ or $-\pi$ exactly, will be considered as equivalent. Nevertheless, pauses and flow variations within the respiratory cycle are well reproduced by instantaneous phases as can be seen by the deviations from a straight sawtooth pattern for the flow phases in the bottom panel of Fig. 7b.

### Reconstruction of respiratory signals from ECG

To derive measures B1, B2, B3, and B5, the ECG data were processed with the software LibRasch^36^ to identify QRS complexes. We visually verified and manually checked QRS classifications (normal, ventricular ectopic, and supra-ventricular ectopic) and corrected them if necessary. Noisy parts where no QRS detection was possible were manually marked and excluded from further analysis. All normal QRS complexes were used for B1, B2, and B5, while only time intervals between two successive normal QRS complexes were used for B3. The resulting time series were homogeneously resampled at a rate of 4 Hz by cubic spline interpolation. Subsequently, a FFT band pass filter with limit frequencies 0.01 Hz and 0.5 Hz was applied to eliminate variations clearly outside the respiratory band. As an example, the green curve in Fig. 7a shows the B1 proxy for a typical subject during sleep.

### Phase synchronization and comparison of respiratory rates

In order to compare and test two reconstructed respiratory phases $\phi _x(t)$ and $\phi _y(t)$ (for $x,y={\mathrm{Acc}}_x$, ${\hbox {Acc}}_y$, …, flow(*t*)), we calculate the phase synchronization index $\gamma$ by^37^

5 $$\begin{aligned} \gamma (k) = \left| \left\langle \exp [i (\phi _x(t) - \phi _y(t))] \right\rangle \right| , \end{aligned}$$

where the average $\langle \ldots \rangle$ runs over all times from $t-15 \hbox {s}$ to $t+15 \hbox {s}$ with $t=k\cdot 30s$ and *k* is the index of the 30 s windows. This definition has the advantage that constant phase differences between the two proxies (and differences by multiples of $2 \pi$) as well as changing proxy amplitudes are disregarded. A $\gamma$ value close to one indicates strong phase synchronization, i.e., a close similarity of the two phase signals, while a $\gamma$ value close to zero indicates dissimilarity. The approach will be used for comparisons of two proxies as well as comparisons of proxies with the flow signal considered as a reference for real respiration. As examples for the typical values of $\gamma$, we note that the first (second) 30-s window of the signals presented in Fig. 7b yields $\gamma = 0.35$ (0.57) for the comparison of EDR (the B1 proxy, green) with the flow (blue), and $\gamma = 0.98$ (0.90) for the comparison of the ${\hbox {Acc}}_y$ proxy (red) with the flow.

We note that we used respiratory phases derived from the PSG-recorded flow signal as reference without a validation in this study. However, this approach does not lead to any bias regarding the comparison with different respiration proxies, since a corrupted flow signal will not be synchronized with any respiration proxy. Excluding subjects with partly unreliable flow recordings would probably have led to somewhat larger group averages of the phase synchronization index $\gamma$ for all proxies. But since it would also have led to excluding subjects with nocturnal breathing disorders, we have decided against this. Nevertheless, for a subset of 118 PSG recordings, we compared the flow-derived respiratory phase signal with respiratory phases derived from thorax and abdomen inductive plethysmography by calculating the average synchronization indices according to Eq. (5) for each of the three pairs. Our results of $\gamma = 0.68 \pm 0.19$ (comparison flow versus thorax plethysmography), $0.42 \pm 0.33$ (flow versus abdomen plethysmography), and $0.45 \pm 0.34$ (thorax versus abdomen plethysmography) indicate that (i) phase synchronization indices $\gamma$ in the range from 0.4 to 0.7 indicate good phase synchronization and (ii) flow and thorax inductive plethysmography recordings are probably more reliable than abdomen recordings.

In another approach to compare the respiration proxy signals, we calculated and compared respiratory rates. A breathing interval was defined by jumps of the instantaneous respiratory phase $\phi _x(t)$ from a value above + 1 to a value below $-1$ one time step (0.25 s) later, see Fig. 7b (for $x={\mathrm{Acc}}_x$, ${\hbox {Acc}}_y$, …, flow(*t*)). We calculated the average respiratory rate for each 30 s window, and finally obtained an average respiratory rate of all windows for each respiration proxy and the flow signal. We note that this approach defines the beginning of a breath by the phase jump, however, since we only count number of breaths in large windows of 30 s, different beginnings for different proxies are not relevant.

Since the distributions of both, $\gamma$ values and respiratory rates, are close to Gaussian, we applied a two-tailed Student’s t-test to check for the significance levels of differences between our results for all proxies. In addition, we checked for the significance of differences between two sets of similarly sized subgroups, (i) males and females and (ii) subjects with and without diagnosed sleep apnea syndrome; see Table 2 for the numbers of subjects in these subgroups.
